# Malnutrition screening and treatment in pediatric oncology: a scoping review

**DOI:** 10.1186/s40795-022-00643-3

**Published:** 2022-12-22

**Authors:** Jessica Franke, Chris Bishop, Daniel V. Runco

**Affiliations:** 1 Marian University College of Osteopathic Medicine, 3200 Cold Spring Rd, Indianapolis, IN 46222 USA; 2 Department of Pediatrics, Indiana University School of Medicine, 705 Riley Hospital Drive, ROC Suite 4340, Indianapolis, IN 46202 USA; 3 Department of Pediatrics, Division of Hematology/Oncology, Riley Hospital for Children at Indiana University Health, Indianapolis, IN USA

**Keywords:** Nutrition, Malnutrition, Pediatric, Cancer cachexia

## Abstract

**Background:**

Malnutrition and cachexia during pediatric cancer treatment worsen toxicity and quality-of-life. Clinical practice varies with lack of standard malnutrition definition and nutrition interventions. This scoping review highlights available malnutrition screening and intervention data in childhood cancer and the need for standardizing assessment and treatment.

**Methods:**

Ovid Medline, CINAHL, and Cochrane Library were searched for studies containing malnutrition as the primary outcome with anthropometric, radiographic, or biochemical measurements. Secondary outcomes included validated nutritional assessment or screening tools. Two authors reviewed full manuscripts for inclusion. Narrative analysis was chosen over statistical analysis due to study heterogeneity.

**Results:**

The search yielded 234 articles and 17 articles identified from reference searching. Nine met inclusion criteria with six nutritional intervention studies (examining appetite stimulants, nutrition supplementation, and proactive feeding tubes) and three nutritional screening studies (algorithms or nutrition support teams) each with variable measures and outcomes. Both laboratory evaluations (albumin, prealbumin, total protein) and body measurement (weight loss, mid-upper arm circumference) were used. Studies demonstrated improved weight, without difference between formula or appetite stimulant used. Screening studies yielded mixed results on preventing weight loss, weight gain, and survival.

**Conclusion:**

Our review demonstrated a paucity of evidence for malnutrition screening and intervention in pediatric cancer treatment. While a variety of malnutrition outcomes, interventions, and screening tools exist, nutritional interventions increased weight and decreased complications. Screening tools decreased malnutrition risk and may improve weight gain. Potential age- and disease-specific nutritional benefits and toxicities also exist, further highlighting the benefit of standardizing malnutrition definitions, screening, and interventions.

## Background

Pediatric cancer is the leading cause of non-accidental childhood death in the United States with up to 80% of children experiencing malnutrition during cancer treatment [1]. Proper nutrition is fundamental to appropriate growth and development through childhood and adolescence [2]. More importantly, when children with cancer experience malnutrition during treatment, they experience more treatment-related toxicity. Quality of life is diminished due to increased fatigue with effects during treatment, after treatment, and even after the patient is in remission [3]. The effects of poor nutrition are further magnified by potentially delaying or decreasing curative delivery [4–6]. Patients with malnutrition have been identified to tolerate treatment less than patient’s without malnutrition causing dose reductions and treatment delays due to decreased health [5]. Chemotherapy, radiation, surgery, and immunotherapy directly result in nausea, vomiting, and anorexia or other metabolic changes such as weight or muscle loss which are further exacerbated by malnutrition [4, 5]. Malnutrition during cancer treatment can lead to increased deleterious side effects due to compromised immunity leading to higher infection rates, worse physical function, more neuropathy, and overall detrimental effects on quality of life [7–9]. Survival impact of malnutrition alone is challenging to quantify, but individual studies suggest lower survival for patients with poor nutrition [5].

Nutritional screenings for hospitalized pediatric patients are variable among geographic regions and hospitals with pediatric specialists and expertise. Many hospitals have individual, unique protocols for malnutrition screening and intervention. This results in diagnosis data that is difficult to compare and generalize and different thresholds for what nutrition supplementation to initiate and which criteria to base it on [10]. While professional organizations such as the American Society for Parenteral and Enteral Nutrition (ASPEN) and the Children’s Oncology Group (COG) have suggested decision trees for appetite stimulants, temporary nasoenteral feeds, or more durable percutaneous gastrostomy or jejunal feeds in both adult cancer patients and critically ill children, the use of such decision tools remains under-utilized, further contributing to lack of standardized nutrition support [11–13]. These decision trees highlight screening for weight loss, loss of growth velocity, and assessing gut absorption with the ability to tolerate enteral nutrition before proceeding to parenteral or post-pyloric feeding [14]. For adults with cancer, treatments for malnutrition are more standardized, with more readily available oral supplements, parenteral nutrition, and enteral feeding [15]. In comparison, nutritional treatments for pediatric patients may be more difficult because protein and calorie needs change with the growing child making standardization difficult and more research needed to identify beneficial interventions and assessments at these variable time points in a child’s development [16]. Additionally, the use of ideal body weight, normalized weight-for-height and weight-for-age z-scores, and bone and muscle density are recognized as increasingly important [13].

This review aims to summarize and compare evidence-based studies of screening and nutritional intervention for children with cancer. The necessity of proper nutrition during pediatric cancer treatment is crucial to improving toxicity from cancer treatment and potentially survival. Ultimately, this review will be helpful in standardizing protocols for effectively and accurately assessing and treating malnutrition.

## Methods

PICO Criteria was utilized to create a research question and a focused systematic database search (Table 1) [17]. The original search included the electronic databases Ovid Medline, CINAHL, and Cochrane Library. No time limitations were placed on the search due to the very small number of pediatric nutrition studies that were identified. MeSH terms searched included cachexia, neoplasms, carcinoma, radiotherapy, immunotherapy, appetite, appetite regulation, malnutrition, nutritional status, weight-loss, body mass index, body weight, body composition, anthropometry, child nutrition disorders, nutrition assessment, Wilms tumor, and precursor cell lymphoblastic leukemia-lymphoma. Additional search terms included truncated forms of the following keywords: carcinoma, chemotherapy, radiation therapy, tumor, malignant, cancer, neoplasm, oncology, malnutrition, and appetite. Duplicate entries were removed after exporting to EndNote. External sources were hand-searched based on the references from selected articles, and applicable articles were added to the pool of the database search results.

**Table 1:** PICO Criteria for Guided Scoping Review(17)

Research question	In children with cancer undergoing treatment, what interventions or screening methods decrease the incidence or severity of malnutrition as measured by laboratory or body measurements?
Population	Pediatric patients (humans), less than 20 years, with cancer undergoing chemotherapy, radiotherapy and/or immunotherapy treatments • Inclusion: children under 20 years undergoing chemotherapy, radiotherapy and/or immunotherapy • Exclusion: adult or animal studies, observational or non-intervention studies
Interventions	Weight loss treatments • Inclusion: nutrition interventions and cachexia screening tools • Exclusion: studies without nutrition as primary outcome
Comparison, Outcomes	Malnutrition and interventions (nutrition interventions and cachexia screening tools) • Primary outcomes: malnutrition (body measurements, radiographic, biochemical, etc.) • Secondary outcomes: validated nutrition assessment or malnutrition screening tools

Abstracts from the database search were independently and separately reviewed based on the inclusion criteria in Table 1 by two authors before comparing. Discrepancies were discussed and resolved based on the inclusion criteria. Full manuscript review was performed for included abstracts with a final determination made and agreed upon by two authors. The primary outcome for the articles included was the objective measurement of malnutrition in terms of body measurements, radiographic measurements, and biochemical measurements based on research demonstrating correlation between these measurements and malnutrition information [16, 18]. Secondary outcomes included validated nutritional assessment or malnutrition screening tools. Due to heterogeneity in the reported data and low numbers of published studies, no statistical analyses were performed similarly to previously published reviews on nutrition in pediatric cancer care [19, 20]. We developed an approach aiming to complete a systematic review. However, given the heterogeneity and inconsistency of the studies, we designed a scoping review because we were unable to perform a statistical analysis on the data collected. A scoping review by definition is a collection of key concepts of articles within a specific topic along with their location. The results of each study were compared in a narrative manner to reach conclusions and no formal risk of bias analysis was completed. Due to limited and heterogeneous pediatric cancer nutrition studies, this review was not registered.

## Results

**Fig. 1:**
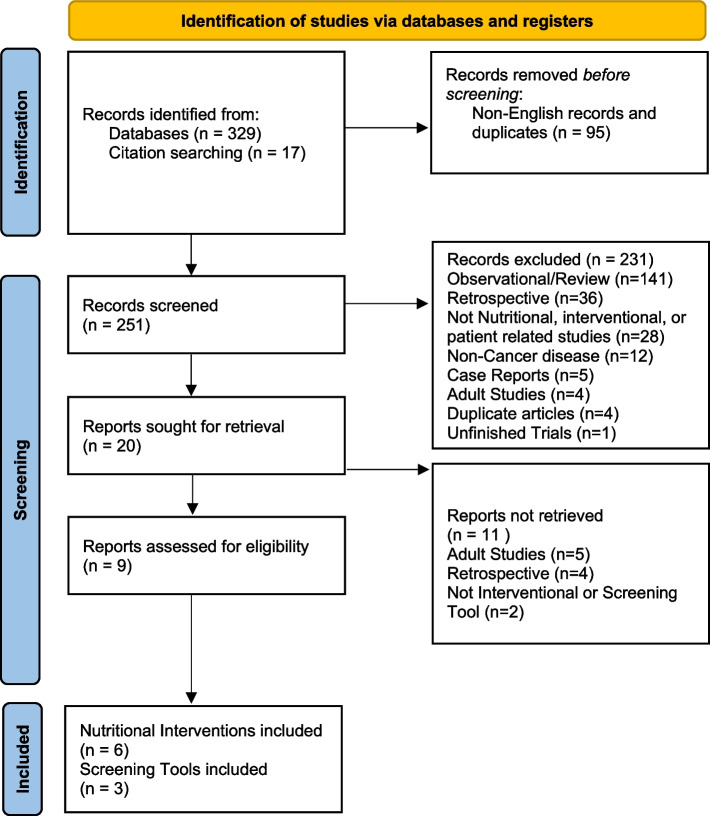
PRISMA 2020 flow diagram for new systematic reviews which included searches of databases and registers only. A*dapted From:* Page MJ, McKenzie JE, Bossuyt PM, Boutron I, Hoffmann TC, Mulrow CD, et al. The PRISMA 2020 statement: an updated guideline for reporting systematic reviews. BMJ 2021;372:n71. https://doi.org/10.1136/bmj.n71

The systematic search yielded 234 papers (Fig. 1). In addition, 17 articles were found from reference searching. A total of 251 article abstracts were reviewed. 231 were excluded (criteria listed in Fig. 1) resulting in 20 articles for full length review. Of the 20 articles fully reviewed, 9 met inclusion criteria (Table 1). Of the included articles, 6 studies described nutritional interventions (Table 2) with the remaining 3 describing implementation or validation of nutritional screening tools (Table 3).

**Table 2:** Included Studies– Nutritional Interventions

Publication	Design or sample*	Measures	Results
Prasad, et.al (2021) [26]	Randomized, open-label phase 3 trial Ready-to-use therapeutic food (RUTF) 260 patients (intervention group *n* = 130; control group *n* = 130)	Biometrics: weight, nutritional status, fat mass Complications: infection, mucositis	• Intervention increased weight gain (77.8% vs 64.2%) (*p* = 0.025) • Significant increase in fat mass (*p* = 0.005) • Increased number of patients with normal nutritional status (*p* = 0.02) • Decreased complications (infections: p < 0.0001; mucositis: *p* = 0.006)
Liang, et.al (2018) [21]	Quasi-experimental study Oral formula supplement 127 patients (intervention group *n* = 67; control group *n* = 60)	Biometrics: weight, hemoglobin, total protein, albumin, prealbumin Complications: hypoalbuminaemia, gastrointestinal complications, and infections	• Increase in weight and hemoglobin with formula supplement (p < 0.05) • Formula supplement increased total protein, albumin, and prealbumin (p < 0.001) • Decreased complications in intervention group (p < 0.05) • Fewer blood and albumin infusions for intervention group (p < 0.05)
Gurlek Gokcebay, et.al (2015) [22]	Monitoring children during cancer therapy Isocaloric versus hypercaloric supplements for children with malnutrition 45 total patients (malnourished *n* = 26; hypercaloric supplement *n* = 18; isocaloric supplement *n* = 8)	Biometrics: weight, BMI, WFH, MUAC, TSF, serum albumin, prealbumin, protein Malnutrition criteria (at least 1 of the following): BMI < 5%ile, WFH < 90%ile, TSFT or MUAC < 5%ile, or 5% weight loss	• No statistical difference between hypercaloric and isocaloric formula • Decrease in malnutrition diagnosis with supplement (*p* = 0.006) • At 6 months, formula increased WFH (*p* = 0.003), BMI (*p* = 0.003), TSF (*P* = 0.007), and MUAC (p < 0.001) • Also increased serum albumin levels (p < 0.001) and prealbumin (*p* = 0.005) at 3 and 6 months
Cuvelier, et.al (2014) [23]	Randomized, double-blind, placebo-controlled study Megestrol acetate (MA) 26 patients (intervention group *n* = 13; placebo group *n* = 13)	Biometrics: weight, WAZ, HAZ, BMI-Z, MUAC, TSF Secondary outcomes: body composition, toxicities	• MA associated with significant weight gain (*p* = 0.003), WAZ (*p* = 0.002), BMI-Z (*p* = 0.006), and MUAC (*p* = 0.01) • No significant difference in HAZ or TSF
Sacks, et.al (2014) [24]	Pilot study Proactive enteral tube feeding 53 patients (intervention group *n* = 20; control group *n* = 33)	Biometrics: WFH, BMI, WAZ Secondary outcomes: infection	• Intervention group had less of a loss in WAZ than control group (19% decrease vs. 40% decrease, respectively) from diagnosis to tube feeding initiation (*p* = 0.037) • No p-values were reported for changes in WFH and BMI • No difference in infectious complications
Couluris, et.al (2008) [25]	Open label phase 2 trial Cyproheptadine hydrochloride (CH) and megestrol acetate (MA) for CH failure CH intervention *n* = 66; MA intervention *n* = 6	Biometrics: weight, growth rate, WFH, WAZ, prealbumin, leptin Treatment response (stable or increased weight)	• CH significantly increased weight (*p* = 0.001), WAZ (*p* = 0.001), serum leptin levels (*p* = 0.0004) • 76% treatment response with CH • 5 of 6 patients on MA responded to therapy • No significant difference in prealbumin

*WFH* weight-for-height, *BMI* body mass index, *MUAC* mid-upper arm circumference, *MA* megestrol acetate, *WAZ* weight-for-age z-score, *ALL* acute lymphoblastic leukemia, *TSF* triceps skinfold thickness, *sample included analyzed patients only

**Table 3:** Included Studies – Screening Tools

Publication	Design or sample	Measures	Results
Gallo, et.al (2021) [27]	Quality improvement report (pre and post intervention) Nutritional support team 145 patients (control group *n* = 73; intervention group *n* = 72	Survival, body measurements, hospitalization and treatment characteristics	• Decreased need for antibiotic treatment (*p* = 0.036) • Nutrition support decreased length of treatment (p < 0.001) • No significant improvement in survival or hospital, treatment, and antibiotic days (p > 0.05)
Han, et.al (2021) [28]	Quality improvement report (pre and post intervention) Nutritional screening tool for childhood cancer (SCAN) Intervention group *n* = 267	Biometrics: weight, malnutrition rates Dietitian referral and timeliness	• Improved dietician referral and timeliness (from 36.4% to 85.7%; p < 0.001) • Improved percent weight change, but not significant (*p* = 0.036)
Totadri, et.al (2019) [29]	Validation study SIOP-PODC algorithm 50 patients (intervention group *n* = 25; control group *n* = 25)	Biometrics: MUAC, weight Complications: mucositis, transfusions, febrile neutropenia	• No significant weight increase • Significant increases in MUAC (*p* = 0.02), and oral supplements (*p* = 0.011) • Fewer platelet transfusions in intervention group (*p* = 0.02) • No difference in mucositis occurrence

### Interventions

Liang et al. (2018) evaluated adding Peptamen® supplements to standard of care nutritional support in children with acute lymphoblastic leukemia (ALL) [21]. The study consisted of 127 patients (intervention group *n* = 60; control group *n* = 67). The intervention group received a low-fat diet with 39.3 g of Peptamen in water 3–5 times per day. The control group received a low-fat diet 3–5 times per day. Peptamen® supplements significantly increased the weight and hemoglobin levels in patients after 30 days of chemotherapy. Significantly higher total protein, albumin, and prealbumin at the end of the 30 days of chemotherapy were also present in the intervention group. Fewer complications of hypoalbuminemia, gastrointestinal complications such as weight loss, and infections were noted as well as fewer blood and albumin infusions needed in the intervention group. While the length of hospital stay was not statistically lower in the intervention group, statistically lower hospital costs were seen.

Gurlek Gokcebay et al. (2015) examined the effects of 6 months of isocaloric (standard of care) and hypercaloric nutritional supplements [22]. There were 45 participants with 18 receiving hypercaloric supplements and 8 receiving isocaloric supplements. Malnutrition criteria for this study was based on patients having at least 1 of the following: body mass index (BMI) < 5^th^ percentile, weight for height (WFH) < 90^th^ percentile, tricep skinfold thickness (TSFT) or mean upper arm circumference (MUAC) < 5^th^ percentile, or 5% weight loss. The study showed malnutrition decreased from 31% to 24% with any supplement use (no significant difference between isocaloric and hypercaloric); and there was no significant difference between isocaloric and hypercaloric supplement usage in WFH, BMI, TSFT, and MUAC at 6 months. After 6 months, the following had a significant increase for the intervention group: WFH, BMI, TSFT, and MUAC. There was also a significant increase in serum albumin levels and prealbumin at the 3- and 6-month mark.

Cuvelier et al. (2014) studied the use of megestrol acetate (MA) as an oral appetite stimulant in children diagnosed with cancer that suffered from weight loss, defined as ≥ 5% body weight or a history with anorexia, and compared it to standard of care [23]. Initially, there were 26 participants (intervention group *n* = 13; control group *n* = 13). 10 participants in the control group were able to complete the study. Children given MA had an increase in mean weight of 19.7% compared to baseline (*p* = 0.003). Children treated with standard of care had mean weight loss -1.2%. Weight-for-age z-score (WAZ), BMI z-score, and MUAC also significantly increased in the intervention group (*p* = 0.01). There was no significant difference in height-for-age z-score (HAZ) or TSFT for the intervention group. In terms of morning cortisol levels, all intervention participants had at least 1 undetectable morning cortisol level while only one participant in the control group experienced similarly low cortisol.

Sacks et al. (2014) studied proactive placement of feeding tubes for children with acute myeloid leukemia (AML) or myelodysplastic syndromes (MDS) [24]. There were 53 patients enrolled in the study (intervention group *n* = 20; control group *n* = 33). The intervention group (those with proactive feeding tube placement) was compared to the control group (those receiving standard of care treatment and diet). The decrease in WAZ was significantly less from time of diagnosis to initiation of tube feeding in the intervention group (19% decrease) compared to the control group (40% decrease) (*p* = 0.037). Proactive feeding tube placement also resulted in less weight loss than the control group.

Couluris et al. (2008) studied 70 patients comparing cyproheptadine hydrochloride (CH) versus MA in children diagnosed with cachexia to determine if treatment could prevent further cachexia (CH *n* = 66; MA *n* = 6) [25]. Documented cachexia was a requisite for enrollment in the study, defined as weight loss ≥ 5%, drop in growth rate two or more percentile ranks on standard growth charts, or a weight-for-height < 10^th^ percentile on standard growth charts. Weight maintenance or weight loss less than 1 percentile was considered a patient response to the supplement. Of the patients given CH (dose of 0.25 mg/kg/d), 76% showed a response with the majority (48/50) gaining weight after 4 weeks (*p* = 0.001). WAZ also significantly increased with CH intervention, and serum leptin levels significantly increased for the CH group on average from 1.19 mg/dL to 1.83 mg/dL (*p* = 0.0004). Serum prealbumin levels did not statistically differ after 4 weeks of therapy. A unique finding of this study was that there was a statistically significant difference among patients with hematologic malignancies (response rate of 91.30%) compared to patients with nonhematologic malignancies (response rate of 67.44%). Patients older than 9 years of age typically gained more weight than those under 9 years of age. 5 out of 6 patients responded to MA therapy with an average weight gain of 2.5 kg.

Prasad et al. (2021) studied the use of ready-to-use therapy food (RUTFs) compared to standard nutritional therapy (SNT) in 260 patients (intervention group *n* = 130; control group *n* = 130) for 6 weeks [26]. The experimental group used RUTFs to meet 50% of their caloric requirement and SNT for the other 50%. The control group was on a strict SNT diet. There was a statistically significant difference in weight gain between groups at 6 weeks (*n* = 126) with a weight gain > 10% in 77.8% of the RUTF group and 64.2% in the SNT group causing more children in the RUTF group to reach a normal nutritional status (based on BMI and MUAC). The RUTF group also had a significant increase in fat mass, and there was a significant difference between groups among children with ALL. 79% of children with ALL in the RUTF group had a weight gain > 10% at 6 weeks compared to 56% of children with ALL in the SNT group. A significant weight gain was not observed in groups of other types of cancer comparing the RUTF to SNT. The RUTF group also had fewer complications: infections (4% vs 19%, p < 0.001) and mucositis (7.9% vs 17.4%, *p* = 0.021); however, both groups showed similar statistics in death due to toxins.

### Screening Tools

Gallo et al. (2021) evaluated the effects of adding a nutritional support team (NST) to aid 73 patients following CNS tumor diagnoses compared to 72 patients that served as a control group [27]. The addition of the NST’s regular screening and therapy decreased malnutrition risk of patients from 65.3% to 32.6% indicating that more patients received nutritional therapy adequate at preventing malnutrition. The length of cancer treatment also decreased significantly but the use of chemotherapy (days) and antibiotics (days) did not statistically differ between groups. Patients treated by the NST team were more likely to reach a 4-year survival (35.6% vs. 25.0%); however, this was not statistically different.

Han et al. (2021) implemented the nutritional screening tool for childhood cancer (SCAN) which significantly improved the frequency of dietician referral in patients at high risk of malnutrition [28]. After conducting a root-cause analysis, the study team identified malnutrition screening as a need for their patient population and implemented the use regardless of patient diagnosis. The study consisted of 267 patients. The study also utilized the PDSA cycle model (Plan, Do, Study, Act) with two improvement cycles to better implement the tool. The dietician referral occurred at a much faster rate (3 days post admission compared to 1 day). Earlier dietitian intervention led to significant increase in weight gain at 1 month and 3 months. Overall, SCAN improved the percent weight change in patients.

Totadri et al. (2019) analyzed 50 patients with cancer (intervention group *n* = 25; control group *n* = 25) [29]. The intervention group consisted of validating a 3-month algorithm tool for determining when to initiate oral supplements, NG feeding, and parenteral nutrition based on the patient’s MUAC and physical health (wasting or eutrophic/overweight/obese). Weight measurements were monitored every 2 weeks. Based on the algorithm, intervention began if the patient was wasting and had a MUAC < 5^th^ percentile. For severe wasting, NG feeding was started. In moderate wasting with a MUAC < 5^th^ percentile, patients took oral supplements for 2 weeks. If patients did not have a significant weight gain, NG feeding was started. Eutrophic, overweight, or obese patients with MUAC > 5^th^ percentile had no intervention unless they began wasting and met the prior criteria. There was no significant difference in weight gain or mucositis occurrence between the two groups. Compared to the non-interventional control group, the experimental group had a greater MUAC median increment and received fewer platelet transfusions and more oral supplements.

## Discussion

Currently, there is a dearth of nutrition-based studies in pediatric oncology patients. Existing studies are primarily retrospective and secondary analyses nested within larger or therapeutic studies. We were able to identify nine prospective, interventional nutrition-focused studies. Of these, six included prospective, nutrition-based interventional studies and three involved validation or outcomes related to nutritional screening. Despite variation in study design and outcomes of interest, the overall findings suggest nutritional interventions increase weight and decrease complications during pediatric oncology treatment while nutritional screening decreases risk for malnutrition and potentially improves weight gain.

Of the studies reviewed for nutritional intervention, five of the six examined the addition of nutrition supplementation including appetite stimulants and various formula compositions. Only one study aimed at proactive enteral tube placement as an intervention. This significant variability in the research surrounding nutrition in children with cancer mirrors the inconsistency in nutritional screening and intervention in clinical practice [16]. While all the studies demonstrated improved weight, there was no significant difference seen in isocaloric versus hypercaloric formulas or ready-to-use therapeutic food versus traditional formulas. This potentially supports the notion that early and appropriate correction of malnutrition can have health benefits, including weight gain or decreased weight loss, regardless of the type of nutritional supplementation used. Similarly, Couluris et al. and Curvelier et al. both observed that cyproheptadine hydrochloride and megestrol acetate both have the potential to lead to improved weight gain compared to placebo [23, 25]. Gokcebay et al. demonstrated that iso-caloric and hyper-caloric supplementation increase serum albumin and prealbumin indicating both interventions decreased malnutrition [22]. Current literature lacks a systematic, evidence-based and patient-centered approach into how and which appetite stimulants or nutrition support tools should be utilized in specific patients, diseases, or treatments. Additionally, the complexity of nutritional intervention to prevent malnutrition is hard to distinguish from support following a diagnosis of malnutrition. Regardless, proactive nutritional intervention including nasogastric tube placement, has been shown to be safe, feasible, and effective [24]. This study suggests pre-empting malnutrition is more effective than treating malnutrition once it has developed.

Previous literature has suggested there are treatment- and disease-specific risk factors for malnutrition, and our study identified interesting findings based on the type of pediatric cancer. Couluris et al. found that patients with hematologic malignancy had improved weight gain on cyproheptadine hydrochloride compared to patients with non-hematologic malignancy also on cyproheptadine hydrochloride [25]. Also, Prasad et al. found greater response to ready-to-use therapy food by patients with ALL compared to standard nutritional therapy [26]. These two observations suggest appropriate interventions may be disease- and treatment-specific. Existing pediatric oncology literature emphasizes the importance of risk-based monitoring for toxicity including increased rates of ototoxocity and hematologic toxicity in teenagers with brain tumors along with more nausea, vomiting, and anorexia in different aged patients treated for lymphoma and rhabdomyosarcoma [30, 31]. Age and treatment intensity have also been shown to impact the risk of developing malnutrition, but we continue to lack prospective interventional studies or widely utilized tools for nutrition screening and intervention in pediatric oncology [32, 33]. Furthermore, in the development of novel therapeutics, documentation shows that changes in weight, specifically body composition, can alter the pharmacokinetics and pharmacodynamics of chemotherapy metabolism [6]. Specific and dedicated study of nutritional interventions directed towards age, disease, and treatment are essential and require further evaluation. 

Examining nutrition screening tools in pediatric oncology yielded even fewer studies. Despite multiple professional societies advocating for systematic and consistent nutritional screening, it remains underutilized in clinical practice and fails to account for the unique medical needs and physiologies of children and adolescents compared to adults [12, 34, 35]. Gallo et al. used implementation of a nutritional support team to decrease the risk for malnutrition and the length of cancer treatment [27]. Nutritional support team implementation also increased 4-year survival rates in patients, specifically with CNS tumor diagnoses. Han et al. found that the SCAN system improves dietician referral and increased weight gain [28]. Totadri et al. studied SIOP-PODC algorithm, which did not increase weight gain [29]. However, it did increase MUAC and supplement usage while decreasing platelet transfusions without decreasing other complications. Overall, the addition of a screening tool benefits patients by preventing weight loss or causing weight gain; but there are several important factors that have not been included in prospective studies. Data demonstrates that nutrition and feeding create significant anxiety in parents and caregivers [36, 37]. It also stands to reason that failure to lose weight or failure to gain weight may reflect earlier intervention and proper nutrition maintenance throughout. Additionally, inclusion of an improved study on nutrition screening could have impacts on quality of life and cancer survivorship. Adult oncologists have better incorporated appetite, body measurements, and function into “cancer cachexia,” but we have even less classification of the cancer cachexia phenotype in children [7, 38].

This scoping review focuses on finding malnutrition interventions and screening tools that adequately treat or prevent cancer-related cachexia. The following limitations should be acknowledged when reviewing the results. First, the frequency of cancer within the pediatric population is far less than the frequency within the adult population. This yields fewer studies regarding cancer cachexia for this scoping review. Second, due to the small number of articles present at the time of this study, the variables reported amongst each study were heterogeneous in comparison to one another. The heterogeneity of variables prevented an accurate meta-analysis from being conducted causing the findings to be conceptual instead of statistical. This is similar to previous cancer related nutritional reviews amongst pediatric patient care that have identified specific challenges in supporting nutrition in children with brain and non-CNS solid tumors specifically [19, 20, 39]. This narrative style of a review is the primary limitation to the study because it does not provide statistics to aid in standardization of care. Lastly, there is variability in treatment appropriateness within the pediatric population due to the vast developmental differences between the youngest and oldest patients within this population. The variability of needs within the pediatric population should not be neglected when looking at these results because developmental variability caused varying intervention and screening results based on age. This review intended to be as inclusive as possible amongst the pediatric population with cancer and cancer related cachexia without widening the scope past the review’s purpose.

## Conclusion

This review highlights the few prospective, interventional trials for pediatric malnutrition screening and intervention that exist for children undergoing cancer treatment. Wide variability in assessment tools, target outcomes, and interventions make determinations about clinical effectiveness difficult. The inconsistency in defined outcomes of interest also limit the ability to standardize research in this field without more prospective nutritional intervention studies in pediatric patients treated for cancer. Specifically, a critical need exists for randomized control trials examining the independent effect of nutrition alongside therapeutic studies. While it is challenging to do isolated nutrition studies, incorporating upfront nutritional intervention in pediatric clinical trials is a potential area of focus. Standardizing malnutrition screening and nutritional intervention and supports will be vital in continuing to improve our cancer-directed therapies for children while minimizing toxicity and improving survival.

## Data Availability

N/A.

## Abbreviations

AML: Acute myeloid leukemia
ASPEN: American Society for Parenteral and Enteral Nutrition
BMI: Body mass index
CH: Cyproheptadine hydrochloride
HAZ: Height-for-age z-score
MA: Megestrol acetate
MDS: Myelodysplastic syndromes
MUAC: Mean upper arm circumference
NST: Nutritional support team
PDSA: Plan, Do, Study, Act
RUTFs: Ready-to-use therapy food
SCAN: Nutrition screening tool for childhood cancer
SNT: Standard nutritional therapy
TSFT: Tricep skinfold thickness
WAZ: Weight-for-age z-score
WFH: Weight for height
